# Adaptation of medical laboratory scientists to workplace hazards – experiences from the COVID-19 pandemic

**DOI:** 10.3389/fpubh.2022.997049

**Published:** 2022-09-28

**Authors:** Blanka Wolszczak-Biedrzycka, Anna Bieńkowska, Stanisław Maksymowicz

**Affiliations:** Department of Psychology and Sociology of Health and Public Health, School of Public Health, Collegium Medicum of the University of Warmia and Mazury, Olsztyn, Poland

**Keywords:** medical laboratory scientists, adaptation, COVID-19, work environment, work in pandemic

## Abstract

The COVID-19 pandemic has dramatically changed healthcare personnel's working environment and sense of security. Medical laboratory scientists were also faced with new occupational challenges. They were tasked with performing novel tests for SARS-CoV-2 without being aware of the associated risks. At the beginning of the pandemic, strict sanitary requirements and the fear of becoming infected with the “new virus” were considerable sources of stress. However, these stress responses abated over time. The aim of this two-stage study was to explore the extent to which this group of medical professionals adapted to new working conditions 1 year after the outbreak of the pandemic. The study was conducted at the beginning of the fourth pandemic wave in Poland, i.e., between 10 September and 31 October 2021. The first stage was a pilot study that involved interviews with 14 medical laboratory scientists. The results were used to perform a survey of 294 laboratory scientists in the second stage. The study investigated the problems and fears faced by this professional group at the beginning of the pandemic, as well as changes in their attitudes during successive waves of COVID-19. The analyzed data demonstrated that most medical laboratory scientists had grown accustomed to the pandemic and workplace changes by the beginning of the fourth wave. The study also indicates that in addition to adequate means of personal protection, health professionals should also be provided with emotional support in times of pandemic.

## Introduction

In late 2019, the world's eyes were on China and the increasingly worrying reports on the emergence of a new severe acute respiratory syndrome coronavirus 2 (SARS-CoV-2). It soon became apparent that SARS-CoV-2 was the cause of the global COVID-19 pandemic which paralyzed the entire world (1, 2). The first case of SARS-CoV-2 infection was confirmed in Poland on 4 March 2020. As of that date, medical personnel found themselves in a new and uncertain reality that posed numerous challenges. Physicians, nurses, and emergency medical services came into direct contact with COVID-19 patients. Medical technologists and other laboratory personnel were tasked with analyzing biological specimens to identify patients infected with SARS-CoV-2.

The pandemic elicited strong emotions in the medical community. Medical professionals were highly motivated to fight the new enemy, help patients and save lives, but at the same time, they were afraid of becoming infected and transmitting the disease to their families. In addition to psychological stress, medical personnel had to deal with a deficiency of personal protective equipment (PPE), staff shortages, overwhelming fatigue and disinformation. Medical professionals are exposed to pathogens on a daily basis, and they are particularly susceptible to infections. Advanced PPE is needed to create a safe and effective work environment for medical personnel (3). However, according to the official data published by the Polish Ministry of Health, 81,844 nurses, 32,872 physicians, 13,410 physiotherapists, 8,416 midwives, 4,616 pharmacists, 4,116 paramedics, 3,986 dentists, and 3,146 laboratory scientists became infected with SARS-CoV-2, whereas 251 physicians, 201 nurses, 24 midwives, 22 pharmacists, seven physiotherapists, seven paramedics, and five laboratory scientists died due to COVID-19 between the beginning of the pandemic and 5 December 2021, despite the fact that strict protective measures had been put into place (4).

Medical laboratory scientists also bore the brunt of the health disruption caused by the COVID-19 pandemic. Until recently, this relatively small group of medical professionals has been largely neglected in public discourse. Medical technologists performing laboratory analyses remain largely anonymous, and very few people are aware that the results of diagnostic tests influence more than 70% of medical decisions (5). During the pandemic, this group of healthcare workers stepped out of the shadow because medical laboratories were tasked with performing thousands of PCR tests to confirm SARS-CoV-2 infections. The public and the authorities became aware that laboratory personnel play a crucial role in healthcare. Special guidelines were issued for dealing with specimens for SARS-CoV-2 testing, as well as all biological samples collected from patients with confirmed or suspected COVID-19. Similarly to other medical personnel, laboratory scientists became overwhelmed by the immense burden of COVID-19 and rigorous sanitary measures, but they rose to the challenge. This study had been conducted before pandemic restrictions were lifted, but by that time, medical workers' emotional responses to the health crisis clearly differed from their attitudes at the beginning of the pandemic (5, 6).

The main research question was: to what extent have laboratory technologists' attitudes to work-related hazards changed between 11 March 2020 [when the WHO declared the novel coronavirus outbreak a global pandemic (7)] and October 2021 (which marked the beginning of the fourth wave in Poland). We tested the research hypothesis postulating that medical laboratory scientists had gradually adapted to dangerous working conditions during the COVID-19 pandemic. Changes in attitudes toward occupational hazards were analyzed among laboratory scientists who were and were not responsible for testing biological specimens for SARS-CoV-2.

The emotional well-being of medical staff during the pandemic has been widely researched around the world. However, the problems associated with the quality of the work environment, new duties and challenges have not been analyzed in detail to date (8–14). Therefore, the aim of this study was to explore the ways in which medical laboratory scientists adapted to the new reality. The results were used to formulate recommendations for creating a safe work environment in dangerous circumstances.

## Materials and methods

The study was conducted between 10 September and 31 October 2021 in two stages: fist pilot stage (qualitative, based on in-depth interviews) and second stage (quantitative, based on on-line questionnaire).

Mixed methods design was used for the study (15). On the first stage (pilot study) qualitative method – face-to-face in-depth interview - was used to explore and obtain depth of understanding of the research area related to the work of the respondents. The main research questions of the pilot phase and overall study were: (1) what challenges accompanied the work of diagnosticians during a pandemic, and (2) how these challenges changed with the changing situation and progressive adaptation.

On the second stage, quantitative method (on-line questionnaire) were used to test and confirm hypotheses based on the obtained knowledge from the first stage (16).

Medical laboratory scientists employed in both public and private laboratories participated in both stages of the study.

The first stage of the study was a qualitative pilot study that involved 14 laboratory scientists from the Polish Region of Warmia and Mazury. The respondents were selected by purposeful sampling. The research consisted in conducting face-to-face in-depth interviews with selected people. The interviews was based on the standardized questionnaire containing 10 open questions about: type of work, workplace experience at the beginning of the pandemic and on the day of the survey, emotional responses during the pandemic, sense of being appreciated, sense of security, and the main concerns. The interviews lasted from 20 to 45 min. Upon the participants' consent, the interviews were recorded, transcribed and coded following content analysis aimed at identifying the frequencies of data and themes. Obtained responses were divided into categories and the main problems and became categories for the nation-wide quantitative survey. The laboratory scientists interviewed in the first stage of the study are characterized in Table 1.

**Table 1:** Description of the surveyed population in stage 1 – qualitative pilot study (*N* = 14).

		**Stage 1 (*n* = 14)**
Age		28–63 (mean: 42)
Sex	Male Female	2 (14%) 12 (86%)
Education	Master's degree in healthcare analytics Master's degree in biology Master's degree in biotechnology Master's degree in environmental protection	7 (50%) 4 (29%) 2 (14%) 1 (7%)
Workplace	Laboratory in a public hospital Laboratory in a public healthcare facility Laboratory in a private healthcare facility	12 (86%) 1 (7%) 1 (7%)

The participants for the second stage of study (quantitative survey) were selected by purposeful sampling. The subjects received a link to an online questionnaire by e-mail and were asked to forward the link to their colleagues (snowball sampling). A total of 294 medical laboratory scientists participated in the study. The majority of the participants did not have specialty training (67.3%) and were employed in public hospital laboratories (52%). The population investigated in the second stage of the study is characterized in Table 2.

**Table 2:** Description of the surveyed population in stage 2 – quantitative survey (*N* = 294).

Education	Medical laboratory scientist without specialty training Medical laboratory scientist with specialty training Medical laboratory scientist - other	198 (67.3%) 92 (31.3%) 4 (1.4%)
Place of employment	Laboratory in a public hospital Laboratory in a public outpatient facility Laboratory in a private healthcare facility (hospital) Laboratory in a private healthcare facility Other or more than one place of work	153 (52%) 20 (6.8%) 43 (14.6%) 49 (16.7%) 29 (9.9%)

The questionnaire comprised 18 questions in the following categories: demographic data, type and place of work, working conditions, work during the COVID-19 pandemic (battery of 14 subcategories on Likert's scale), psychological support, emotions (battery of seven subcategories on Likert's scale), problems with professional performance during the pandemic, sense of being appreciated by various social groups, perceptions of risk, work-related fears at the beginning of the pandemic and on the day of the survey (two questions with seven subcategories), vaccination, and current challenges.

The obtained data from the nation-wide quantitative survey were processed with the use of the IBM SPSS Statistics 27 software platform. The correlations between the variables were assessed by calculating the Pearson correlation coefficient.

The study was approved by the Research Ethics Committee of the University of Warmia and Mazury in Olsztyn, Poland (approval No. 3/2022).

## Results

### New challenges and adaption

**Figure 1:**
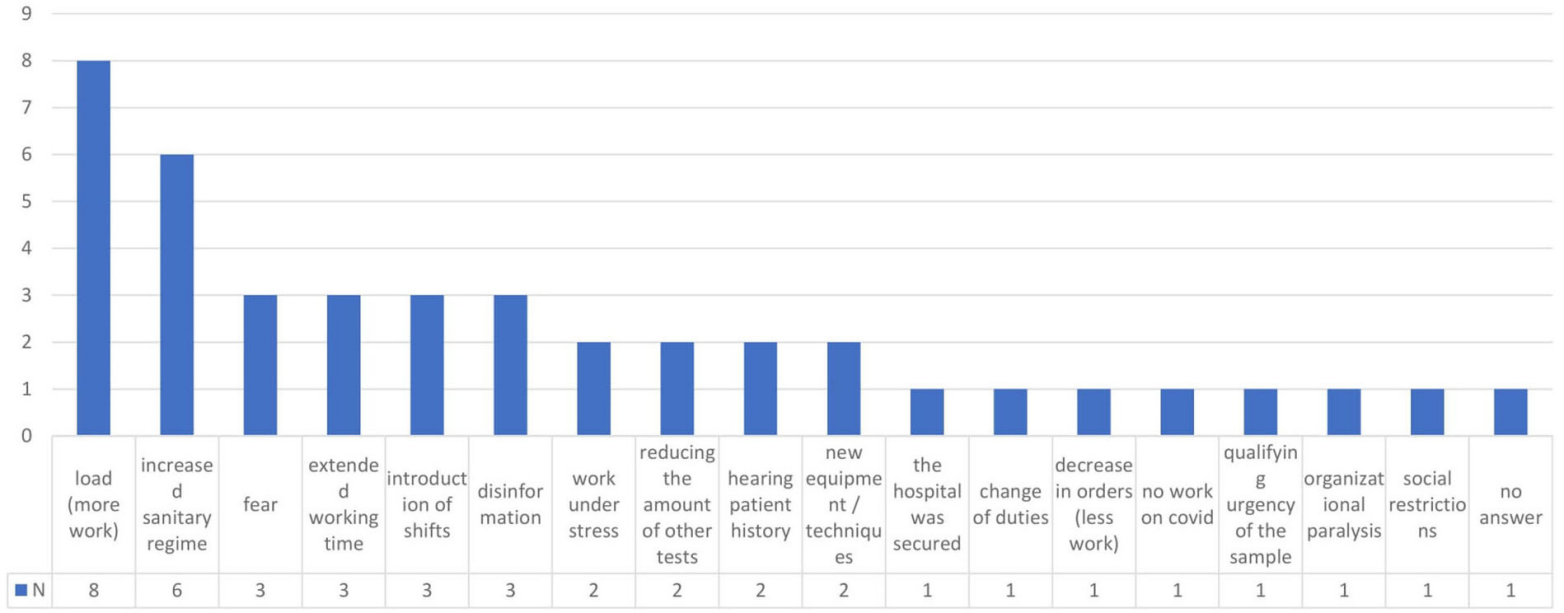
Interview responses regarding the experience from the beginning of the pandemic (***N*** = 14).

The detailed objective of the pilot study was to identify new challenges facing medical technologists during the health crisis. It should be noted that the study was conducted ~1 year after the outbreak of the COVID-19 pandemic. Therefore, the participants were able to reflect on their experiences and emotions at the beginning of the pandemic and compare their initial attitudes and feelings with those reported on the day of the survey. Based on the interviews (1st qualitative stage), the following categories were distinguished as shown in Figure 1.

The categories from the interviews were converted into questions in the quantitative survey. The introduction of a strict sanitary regime at the beginning of the pandemic was recognized as the most severe problem by 95.5% of the surveyed subjects. The lack of reliable information, recommendations and guidelines for medical laboratory personnel was identified as a significant problem by 79.9% of the respondents.

Other adverse consequences of the COVID-19 crisis included: (1) increased workload (84% of the respondents who tested biological specimens for SARS-CoV-2; 69% of the respondents who did not perform such tests), (2) longer working hours (59.2 and 44%, respectively), and (3) additional work shifts (52.5 and 25%, respectively). All of the above factors significantly (***p*** < 0.001) affected medical laboratory scientists who tested biological specimens for the virus, which implies that this group was more influenced by the adverse changes in the workplace.

The health crisis was a major cause of stress, but the studied subjects did not report emotional disorders such as depression, insomnia, or despair. At the beginning of the pandemic, 80.3% of the respondents had a fear of becoming infected or transmitting the virus to their families (86%). However, no significant differences were found between medical laboratory scientists who had and had not tested specimens for SARS-CoV-2.

The study tested the research hypothesis postulating that medical laboratory scientists had gradually adapted to dangerous working conditions during the COVID-19 pandemic. And indeed medical scientists fears and discomfort abated over time. The respondents did not struggle with psychological issues (depression, insomnia, despair) during the pandemic and did not experience intense stress or fear, which could be attributed to the fact that unlike front line medical personnel, most laboratory scientists do not come into direct or prolonged contact with patients with confirmed or suspected COVID-19. It should also be noted that 34% of the respondents had not directly analyzed clinical specimens, i.e., they had not tested respiratory tract samples which are most infectious.

Despite the above, the majority of the surveyed subjects (66%) were of the opinion that psychological assistance would enable them to better cope with work-related stress. However, only 8 out of the 294 analyzed respondents had received such assistance.

**Figure 2:**
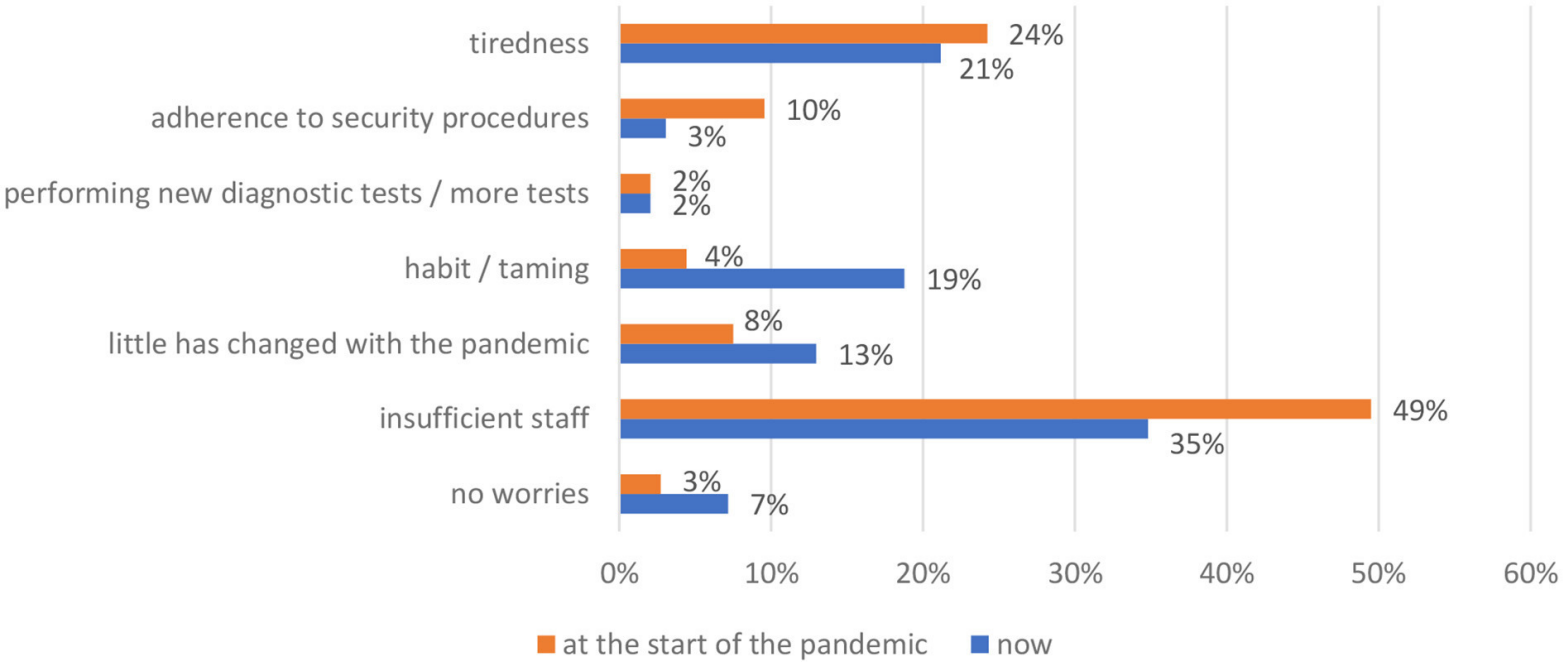
Changes in perceptions of the health crisis since the beginning of the pandemic (*N* = 294).

When asked about their experiences and emotions at the beginning of the pandemic and one year into the crisis, the surveyed scientists were of the opinion that they had largely adapted to the new situation: they reported lower levels of fatigue (decrease of 3%), less stress associated with health safety protocols (decrease of 7%), and staff shortages (decrease of 14%). The percentage of medical laboratory scientists who had adapted to the health crisis increased from 4% at the beginning of the pandemic to 19% on the day of the survey (Table 3; Figure 2).

**Table 3:** Changes in perceptions of the health crisis since the beginning of the pandemic (***N*** = 294).

	**At the beginning**	**At present**
Did not experience fear	3% (8)	7% (21)
Staff shortages	49% (145)	35% (102)
Not much has changed during the pandemic	8% (22)	13% (38)
Became adapted to the new situation	4% (13)	19% (55)
New laboratory tests / increased workload	2% (6)	2% (6)
Safety protocols	10% (28)	3% (9)
Fatigue	24% (71)	21% (62)

### Social recognition and gratitude for medical laboratory personnel

Social recognition of medical laboratory personnel was a frequently raised issue during in-depth interviews in the first stage of the study. According to one of the respondents, “our work is still not appreciated by our superiors or the government.” Medical laboratory scientists have received greater praise from the public, “but we still work in the shadow of other medical professions. People feel more grateful to physicians and nurses” [B.1].

According to the surveyed subjects, the attitudes toward laboratory professionals have changed during the pandemic as more people became aware of their role in combatting the health crisis. “Before the pandemic, most people had no clue about laboratory work. This has changed because during the pandemic, people turned to us for advice about COVID-19 and screening tests” [B.8]. However, many respondents continue to experience a deep sense of injustice: “Our work is not recognized by our superiors, the government or the public. Only we are aware the extent to which our efforts have contributed to ending the pandemic” [B.3]. Similar sentiments were expressed by all participants. One of the respondents said: “I have been praised for my work, and I have been told that the hospital would have to close without our input, and that laboratory technologists are the driving force toward combating the crisis. We received bonuses from the government in recognition of our hard work. When it comes to social gratitude, my family definitely appreciate what I do, and they are proud of me. The medical technologist's profession has gained some recognition during the pandemic. Most people think that lab tests are done by nurses, and perhaps the pandemic has raised awareness levels. Still, this is not enough” [B.9]. However, growing levels of social awareness also prompted some people to fear medical laboratory scientists during the pandemic. “I don't know if our work is more appreciated. Some neighbors who know where I work would run away as soon as they saw me” [B.10].

**Figure 3:**
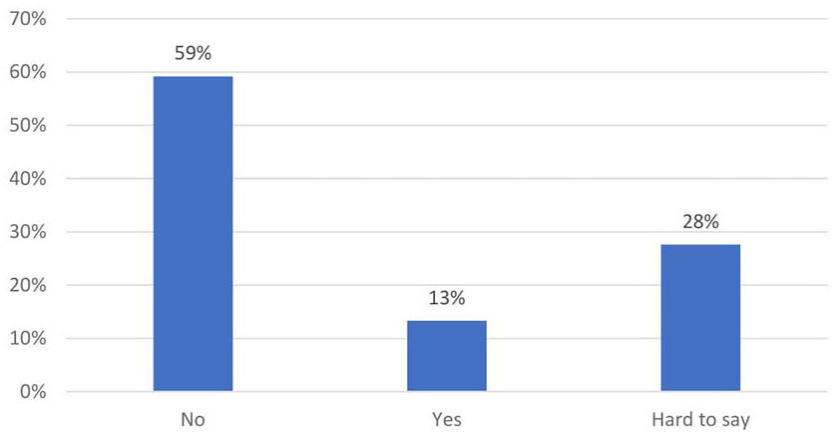
Have you been appreciated by your employer? (*N* = 294).

**Figure 4:**
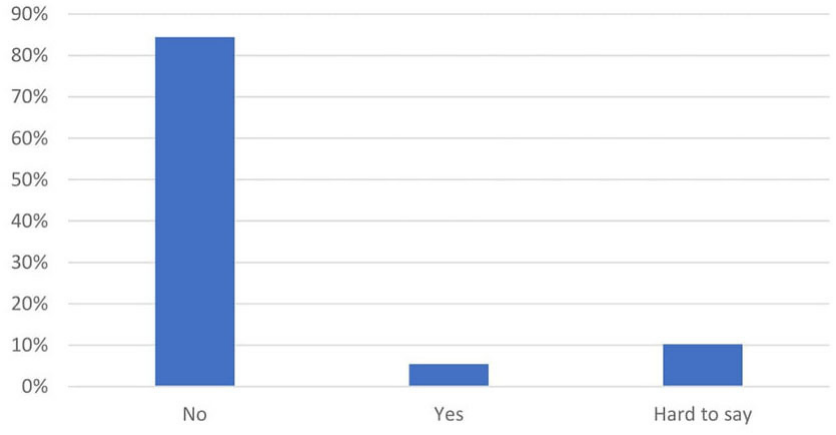
Have you been appreciated by the rulers? (*N* = 294).

**Figure 5:**
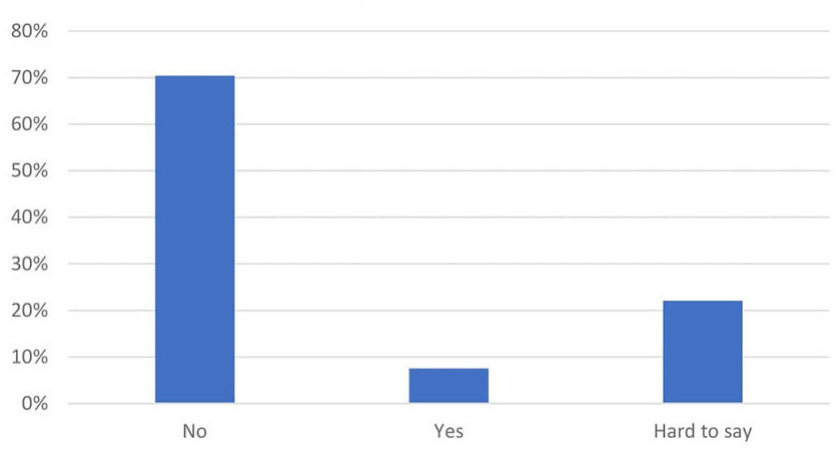
Have you been appreciated by the society? (*N* = 294).

The quantitative survey in the second stage of the study confirmed that laboratory technologists felt largely undervalued (Figures 3–5). When asked whether they had received due recognition from their superiors during the pandemic, 59.2% (174) of the respondents answered in the negative, and only 13.3% (39) replied in the affirmative, whereas the remaining subjects (27.6%, 81) were undecided (“hard to say”). The respondents felt even more neglected by the authorities: 84.4% (248) did not feel appreciated, 10.2% (30) were undecided, and only 5.4% (16) felt appreciated. In the pilot survey, most of the respondents argued that they had received recognition mainly from their public, but these observations were not confirmed in the second survey conducted on a larger population. In the second survey, the majority of medical laboratory scientists claimed that “nothing has changed” (70.4%, 207); 7.5% (22) were of the opinion that their work was more appreciated, whereas the remaining participants (22.1%, 65) were undecided.

At the end of presenting the results, it should be mentioned that one of our initial assumptions has not been confirmed, that work that involved testing biological specimens for the presence of SARS-CoV-2 was the main variable differentiating the examined population. In stage two of the study (nation-wide quantitative survey), most of the participants (66%) had tested biological specimens for the virus, whereas 34% of the respondents had not performed such tests. However, the statistical analysis revealed no significant differences in feelings of discomfort or low mood between these groups. The new situation posed a considerable challenge for both groups of participants who did not differ significantly in their emotional attitudes. The analyzed grouped differed significantly only in their attitudes toward an increased workload, and this problem was significantly (*p* < 0.001, Pearson's correlation) more often reported by medical laboratory scientists who tested biological specimens for SARS-CoV-2. This observation could be explained by the fact that both groups had similar attitudes to the examined problems in an increased sanitary regime. Due to the absence of significant differences, the division of the studied population into two groups was abandoned, and successive analyses were conducted based on descriptive statistics.

## Discussion

This study was conducted to investigate the emotions and fears experienced by laboratory staff at the onset of the pandemic, and the extent to which these feelings changed in the course of the pandemic.

Healthcare professionals, including physicians, nurses, paramedics, and laboratory scientists, are indispensable in the process of treating COVID-19 infections. Medical personnel are more susceptible to COVID-19 than the general population because they come into direct contact with infected individuals and biological specimens collected from patients. Increased workload under immense pressure, fear of infection, and staff shortages contribute to burnout and mental health issues in healthcare professionals (3).

Pappa et al. (10) and Wu et al. (8) conducted meta-analyses of more than 80 research studies performed around the world and reported a growing incidence of emotional problems, including depression, insomnia, despair, and anxiety, in healthcare professionals during the COVID-19 pandemic. These problems were most prevalent in medical employees who came into direct contact with patients, i.e., physicians, nurses, and paramedics (17).

There is a general scarcity of reliable data that could confirm or rule out the risk of infection with SARS-CoV-2 through contact with an infected patient's blood or urine. Viremia was reported in 15% of the first COVID-19 patients in Wuhan, China, but the risk of viral transmission to medical personnel, including medical laboratory scientists, was evaluated as low if adequate security protocols and PPE were applied (18). Genetic and antigen tests for SARS-CoV-2 are performed in laminar flow cabinets, and laboratory technologists wear PPE, which significantly reduces the risk of infection. According to the surveyed medical laboratory scientists, the availability of PPE was low in the 1st months of the pandemic, but it improved gradually over time (13, 18, 19).

The implementation of a strict sanitary regime was a source of significant distress and discomfort for more than 90% of the studied population. Prolonged use of PPE and disinfectants can contribute to allergies, in particular respiratory and skin allergies, allergic conjunctivitis, and acute generalized allergic reactions. These conditions are classified as occupational diseases. Research into the use of PPE during a previous SARS epidemic in Singapore revealed adverse skin reactions in medical personnel wearing N95 masks (35% of users) and protective gloves (21% of users) (20). In a Polish study analyzing hand skin problems in laboratory technologists, 98% of the surveyed subjects reported allergies and rashes after prolonged use of protective gloves and disinfecting agents (21).

Some researchers have argued that the COVID-19 infection caused by SARS-CoV-2 should be classified as an occupational disease resulting from exposure to harmful agents in the workplace or associated with the performed duties. The Occupational Safety and Health Administration (OSHA) of the USA developed a classification system for assessing the risk of exposure to SARS-CoV-2 in the workplace. According to the proposed classification, healthcare workers (including physicians, nurses, dentists, and paramedics) and medical laboratory scientists who collect and/or analyze samples from patients with confirmed or suspected COVID-19 are at very high risk of contracting the disease (22). The described classification system also states that workers at high risk of COVID-19 infection, including physicians, nurses, paramedics, as well as laboratory scientists, should receive psychological support (23–26). These types of solutions have been implemented in some Chinese and Italian hospitals (27). Support schemes targeting not only frontline personnel, but all medical sector employees would also considerably benefit healthcare professionals in Poland. Medical laboratory scientists have adapted to the pandemic, but the risk of new SARS-CoV-2 mutations or pandemics caused by new pathogens causes a lot of uncertainty about the future. It should be noted that the respondents did not worry only about their health and lives. They also voiced concerns about the Polish economy (36.9%), the possibility of successive lockdowns (23.8%), low levels of preparedness in the public healthcare system (21.1%), and new virus mutations. These results indicate that medical laboratory scientists, as well as other healthcare professionals, are not only self-preoccupied and have more cause for concern than the representatives of non-medical professions.

The main strength of this study is that it makes the first ever attempt to analyze medical laboratory workers' adaptation to new working conditions during the COVID-19 pandemic and focuses mainly on their emotional well-being. The present findings probably also apply to other healthcare professionals. It is worth noting that the study involved a nationwide survey. The limitations include the fact that the first stage of the study involved only medical laboratory scientists working in various health care units, but in the same city.

## Conclusions

Medical laboratory scientists have gradually adapted to their work environment in a new reality during the COVID-19 pandemic. According to 34% of the respondents, their sense of security increased in successive months of the pandemic. According to 24% of the surveyed subjects, their anxiety was considerably alleviated by the introduction of the vaccination program at the beginning of 2020. However, nearly 20% of the analyzed laboratory technologists felt “pressured” to become vaccinated. The vaccination rate in this professional group was estimated at 90% (27), which indicates that a large number of laboratory scientists received the vaccine despite personal beliefs.

At the beginning of the fourth wave of the COVID-19 pandemic in Poland, medical laboratory scientists' concerns did not focus solely on their professional duties. They had to deal with strong emotions, as well as concerns about their health and families. At present, they are concerned mainly about the Polish economy. These results confirm that Polish medical laboratory scientists have largely adapted to the new reality.

### Recommendations

Based on the results of the present study, the following recommendations can be formulated to improve the performance of medical laboratory scientists who faced considerable pressure and uncertainty in a difficult and changing work environment during the COVID-19 pandemic:

- training on effective communication with patients to reduce stress and fatigue,
- training on the use of PPE to avoid infection,
- training on the use of computer systems (hospital systems and public systems) related to the COVID-19 pandemic to reduce stress and fatigue,
- psychological assistance to reduce stress,
- changes in working hours, depending on the type of work, to reduce fatigue and avoid infection.

## Data availability statement

The original contributions presented in the study are included in the article/supplementary material, further inquiries can be directed to the corresponding author/s.

## Author contributions

Conceptualization: BW-B and AB. Methodology and data curation: SM. Software, formal analysis, investigation, writing—original draft preparation, and writing—review and editing: BW-B and SM. Validation and resources: BW-B, AB, and SM. All authors contributed to the article and approved the submitted version.

## Conflict of interest

The authors declare that the research was conducted in the absence of any commercial or financial relationships that could be construed as a potential conflict of interest.

## Publisher's note

All claims expressed in this article are solely those of the authors and do not necessarily represent those of their affiliated organizations, or those of the publisher, the editors and the reviewers. Any product that may be evaluated in this article, or claim that may be made by its manufacturer, is not guaranteed or endorsed by the publisher.
